# Immobilization of Dystrophin and Laminin α2-Chain Deficient Zebrafish Larvae *In Vivo* Prevents the Development of Muscular Dystrophy

**DOI:** 10.1371/journal.pone.0139483

**Published:** 2015-11-04

**Authors:** Mei Li, Anders Arner

**Affiliations:** Department of Physiology and Pharmacology, Karolinska Institutet, Stockholm, Sweden; University of Minnesota Medical School, UNITED STATES

## Abstract

Muscular dystrophies are often caused by genetic alterations in the dystrophin-dystroglycan complex or its extracellular ligands. These structures are associated with the cell membrane and provide mechanical links between the cytoskeleton and the matrix. Mechanical stress is considered a pathological mechanism and muscle immobilization has been shown to be beneficial in some mouse models of muscular dystrophy. The zebrafish enables novel and less complex models to examine the effects of extended immobilization or muscle relaxation *in vivo* in different dystrophy models. We have examined effects of immobilization in larvae from two zebrafish strains with muscular dystrophy, the *Sapje* dystrophin-deficient and the *Candyfloss* laminin α2-chain-deficient strains. Larvae (4 days post fertilization, dpf) of both mutants have significantly lower active force *in vitro*, alterations in the muscle structure with gaps between muscle fibers and altered birefringence patterns compared to their normal siblings. Complete immobilization (18 hrs to 4 dpf) was achieved using a small molecular inhibitor of actin-myosin interaction (BTS, 50 μM). This treatment resulted in a significantly weaker active contraction at 4 dpf in both mutated larvae and normal siblings, most likely reflecting a general effect of immobilization on myofibrillogenesis. The immobilization also significantly reduced the structural damage in the mutated strains, showing that muscle activity is an important pathological mechanism. Following one-day washout of BTS, muscle tension partly recovered in the *Candyfloss* siblings and caused structural damage in these mutants, indicating activity-induced muscle recovery and damage, respectively.

## Introduction

Muscular dystrophy is a heterogeneous group of inherited disorders, manifested by progressive skeletal muscle wasting and degeneration [1]. The primary cause of these disorders is often a lack of functional membrane-associated structural proteins in muscle due to genetic defects. The most common and severe form is Duchenne muscular dystrophy (DMD), which is caused by mutations in the dystrophin gene. This protein is an intracellular component of the dystrophin-glycoprotein complex (DGC), a membrane-associated structure providing attachments between the cellular cytoskeleton and the extracellular matrix [2]. Laminin α2-chain (merosin) is an extracellular component, binding to the DGC via α-dystroglycan and attaching to the matrix. In addition, laminin α2-chain has other interactions via integrins (α7β1) in the cell membrane [3]. Mutations in the laminin α2 gene have been shown to cause merosin-deficient congenital muscular dystrophy type 1A, also known as MDC1A [4].

The pathogenesis of muscular dystrophy has been extensively investigated; mechanical stress, oxidative challenge, growth factors and stretch-activated ion channels have been shown to be involved. The DGC complex and the laminin α2-chain binding integrins have major signalling functions in muscle, and alterations in these structures influence several cellular processes affecting muscle development and structure [5, 6], and thus potentially the disease development in muscular dystrophy. The precise mechanism initiating the onset of the dystrophy is still not clear. Given the position of the proteins, both dystrophin and laminin α2-chain are essential for the maintenance of muscle sarcolemmal integrity. It has been established that mechanical damage to the cell membrane is a key pathological mechanism in DMD and it might also play an important role in MDC1A pathogenesis [7–9]. To examine this in an *in vivo* context, different immobilization procedures have been applied to the dystrophin-deficient *mdx* mouse model. Using hindlimb immobilization, Mokhtarian et al. [10] showed that muscle fiber necrosis could be prevented. Other studies have suggested that immobilization can induce apoptosis, muscle weakness and susceptibility to contraction-induced injury in this model [11, 12]. The effect of increased muscle activity and exercise appears to be variable in the *mdx* mouse model, but in a study on diaphragm muscle, it was found that long term wheel running resulted in attenuation of muscle function [13]. *In vitro* studies applying eccentric contractions on muscles from laminin α2-chain deficient C57BL6J/dy^2j^ mice does not support that stretch induced injury at the membrane level is primary mechanism [14]. However, early work on the *dy/dy* dystrophic mouse has suggested that immobilization improved the muscle contraction. In view of the lack of documented membrane rupture in the laminin α2-chain deficient models, the precise pathological mechanism for the effects of immobilization/activity remains to be clarified. It also should be noted that all immobilization studies on dystrophic mice involve comparatively complex interventions with potential effects on whole animal physiology that might affect the muscle-specific changes.

Much of the current knowledge on the disease mechanisms originates from studies of mouse models. For DMD, the *mdx* model mimics some of the characteristics of the human disease, albeit with a milder phenotype [15–17], possibly due to compensation from other proteins and cell regeneration [16, 18]. The mouse models for MDC1A (*dy/dy* and *dy* ^*2J*^ */dy* ^*2J*^) also exhibit muscle dystrophy with muscle fiber necrosis. Interestingly, the *dy/dy* and *dy* ^*2J*^ */dy* ^*2J*^ mice exhibit less membrane injuries as assayed by Evans blue accumulation, in contrasts to the *mdx* mouse muscles [17], which indicates that the two models might differ with regard to the disease mechanism. Mouse dystrophy models can have some limitations, and it might also be important to examine dystrophy models in alternative species. In this context, the zebrafish is an interesting alternative to the mouse, and some aspects of human muscle disease appear to be better reproduced in the zebrafish [19]. In addition, several technical advantages are present in the zebrafish model including e.g. the possibility to comparatively simply manipulate key genes and perform studies cost efficiently and with high throughput. In the zebrafish, the DMD/*Sapje* model with a dystrophin mutation has a more severe phenotype than the *mdx* mouse with regard to the muscle structural and functional alterations [20–22]. Evans blue accumulation in muscle indicates a membrane leakage in the *Sapje* (*Sap*) mutant [20], and these larvae have significant structural damage in the musculature accompanied by a severe contractile failure [21]. The decrease in active force is most likely due to the reduced muscle mass and possibly also a force transmission failure in the muscle tissue. The *Candyfloss* (*Caf*) zebrafish mutants with laminin α2-chain mutation have significant structural changes in the musculature [23] and in a similar manner as for *dy/dy* mice, Evans blue injection did not give indication of membrane rupture [24]. However, the structural damage in the *Caf* zebrafish mutants was shown to be affected by motor activity [24]. The zebrafish models, in particular, the genetically mutated strains, thus introduce novel possibilities to examine mechanisms of muscle disease.

For translation to the situation in human muscle disease, a functional readout regarding muscle contractility in the zebrafish models is essential. We have therefore developed techniques to examine the function of muscle in zebrafish early larvae [19, 21, 25], and recently shown impaired active force generation in the *Sapje* zebrafish strain [21]. This functional impairment and the structural alterations in the *Sapje* muscles could be partially rescued by treatment with Ataluren, a read-through compound inducing a re-expression of dystrophin [21]. Mechanical properties of the *Candyfloss* muscles are not yet characterized.

The aim of the present study was to examine the effects of active contraction on the pathogenesis of dystrophin and laminin α2-chain associated dystrophies, using the *Sapje* and *Candyfloss* mutant zebrafish. Since the latter mutants have not been functionally characterized, we initially examined if they have a contractile dysfunction similar to that in the human disease. To address questions regarding the role of muscle activity in the development of structural and mechanical deficiencies, we established a protocol where the larvae were immobilized *in vivo* using *N*-benzyl-ptoluene sulphonamide (BTS) a small molecule inhibitor of myosin actin-stimulated ATPase activity [26]. In the zebrafish larvae, it was possible to keep the muscles relaxed and the larvae immobile from 18 hours until 4 days post-fertilization with BTS. Using this approach, we examined the impact of early immobilization *in vivo* on the function of muscle and on the disease development in the dystrophin and laminin α2-chain deficient strains.

## Material and Methods

### Zebrafish strains and handling

The dmd/sap^ta222a^ mutant strain (*Sapje*, *Sap*) was obtained from the Tübingen Stock Collection (Tübingen, Germany). These animals carry a mis-sense mutation with a premature stop codon (in Exon 4) in the dystrophin gene at chromosome 2, and lack dystrophin [20]. The lama2/caf^teg15a^ (*Candyfloss*, *Caf*) strain was imported from Prof. Peter Currie’s group at Monash University. These animals have a mis-sense mutation in the laminin α2 gene (*lama2*, homologue of human exon 60), and show disrupted *lama2* expression [24]. The animals were raised and maintained in the zebrafish facility at Department of Cell and Molecular Biology, Karolinska Institutet. Heterozygous pairs were used for breeding, which gives eggs with normal gene expression (wild type, ~25%), heterozygotes (~50%) or homozygotes (~25%) for the *Caf* and *Sap* mutations. Genotyping was performed using derived cleaved amplified polymorphic sequence (dCAPS) analysis as described previously [23]. In principle, genomic DNA is isolated, subjected to PCR using primers amplifying the region with the mutation. Thereafter the PCR products were subjected to restriction enzyme digestion (*Sap*: DraI; *Caf*: Tsp45I), active only on DNA with the mutation. The products were separated on agarose gels and DNA fragments from homozygous samples were fully digested giving one band with smaller size on the gel. Wild type PCR products were not digested (one band of larger size) and heterozygous samples gave two bands. The homozygous mutant dystrophic larvae used in the experiments were identified either by genotyping or birefringence measurements. The heterozygous and wild type animals had similar and normal phenotype[21, 24] and were used as normal controls and are denoted siblings. The larvae used in this study were younger than 6 day post-fertilization (dpf). They were anesthetized using 0.02% MS-222 (Tricaine methanesulfonate; Sigma, St. Louis, MO, USA) in E3 water (in mM: 5 NaCl, 0.17 KCl, 0.33 CaCl_2_, and 0.33 MgSO_4_), prior to euthanasia and mounting for mechanical experiments or for fixation and biochemical and morphological analyses. This study was carried out in strict accordance with the recommendations in the European Guidelines for animal research, national and local regulations. The protocol was approved by the Local Animal Ethical Committee for Animal Experiments in Stockholm (Permit Numbers: N193/14 and N386/11).

### BTS immobilization

*N*-benzyl-*p*-toluene sulphonamide (BTS, Sigma-Aldrich) was dissolved in DMSO to give a stock solution of 50 mM, and then added to the E3 medium to obtain the final concentration of 50 μM. Embryos were manually dechorionated at 18 hours post-fertilization (hpf) and transferred to 50 μM BTS solution in E3 medium. The incubation with BTS, which completely immobilized the larvae, lasted until 4 dpf and followed by washout in E3 medium. DMSO (0.1%) in E3 medium was used as control. Two conditions were examined: (*i*) larvae at 4 dpf following 1 hour wash-out of BTS/DMSO in E3 medium, and (*ii*) at 5 dpf following wash-out of BTS/DMSO and 1 day recovery in E3 medium. Since the animals were selected at an early stage when the structural phenotype had not penetrated, each treated larva undergoing analysis (structural or mechanical) at 4–5 dpf was genotyped as described above to identify the homozygous mutants and their siblings.

### Morphology

The extent of structural changes in the muscle was evaluated using birefringence microscopy essentially as previously described [21, 27]. It has been shown in *Sap* larvae that altered birefringence (dark areas) is correlated with structural damage, as evaluated by Evans blue injection [28]. The birefringence signal is dependent on the regular arrangement of contractile filaments and loss of this feature, reflected in loss of the birefringence signal is referred to as damage in the current paper consistently with previous reports [21, 27]. The larvae were held, oriented flat on the side, under an inverted light microscope between two sheet polarizers oriented at 90° angle relative to each other, making the background dark and birefringent normal muscle tissue light. These birefringence measurements were done without knowing the genotype and the data were grouped retrospectively according to subsequent genotyping. Photographs were taken and the outline of the muscle (between somites number 5 and 30) was traced using a graphics program (Quantity One, Bio-Rad), and the light intensity per area was evaluated as a measure of the amount of normal birefringent muscle tissue. The intensity values were corrected for background.

General morphology was examined using Rhodamine phalloidin staining of actin. BTS treated larvae from each strain were fixed, using 4% paraformaldehyde in phosphate-buffered saline at 4°C overnight. They were stained and subsequently analyzed as whole-mount preparations under confocal microscope as described previously [21]. To evaluate the extent of myofibrillar disorganization (“waviness”) the following procedure was adopted. The length of myofibrils (between myosepta in the trunk muscles) was measured by tracking the cell outline using LSM 510 Meta (V.3.2 SP2, Zeiss Germany) software. In parallel, a straight line connecting the end-end points between the myospta was taken and the corresponding length was measured. The myofibril length was then divided with the length of straight line as an index for myofibril waviness.

### Behavioral analysis

The zebrafish larvae were examined using an automated behavioral analysis system, ZebraLab V3 (ViewPoint Life Sciences Inc) at 4–6 dpf. The larvae were pre-selected and grouped as mutants (*Sapje* or *Candyfloss*) and their healthy siblings under birefringence. They were transferred into 24-well plate held with E3 medium, one larva per well, an allowed to acclimatize for 30 min at 22°C. The swimming pattern was then recorded by the optical system for 2 min. Total active time and large/small movements of each larva were then analyzed automatically.

### Muscle contractile properties

Whole 4–5 dpf larvae were mounted using aluminum clips between a force transducer and a fixed hook in a bath perfused with MOPS buffered physiological solution as described previously [19]. The preparations were mounted at slack length and then stimulated (single twitches) with 0.5 ms duration electrical pulses (supramaximal voltage) at 2 minute-intervals via two platinum electrodes placed on both sides of the preparation. The length was then increased stepwise between the contractions, from the slack length to a length above the maximal for active force. At each length, active force was recorded. The active force at optimal length was used in the subsequent analysis.

### Statistics

All data are presented as mean ± SEM. Statistical analysis was performed using Sigma plot 8.0 for Windows and SigmaStat for Windows 3.0 (Systat Software, Inc., Chicago, US). N numbers refer to the number of examined animals, and all treatment regimes included in the analyses were performed at least 3 times (i.e. on at least 3 separate egg clutches).

## Results

### Effects of BTS treatment on larval mobility and muscle contraction

BTS (50 μM) was applied from 18 hpf, i.e. before spontaneous movements occur, to 4 dpf. During the treatment period, the larvae were fully immobilized and non-responsive to touch. DSMO (0.1%) was used as control regimen and the larvae in this group moved freely and had quick responses to touch at 3–4 dpf. We have previously shown that BTS reversibly inhibits active contractions in larval muscle [25]. In a separate series of experiments on 5 dpf control larvae, we confirmed the acute prompt inactivation by BTS on isolated muscle preparations, stimulated at 2 min intervals to give single twitch contractions. BTS (50 μM) inhibited active contraction *in vitro* to less than 10% in about 15 min (7±1%, n = 3) and the effect was reversible after 30 min washout (recovery to 98±2%, n = 3).

### Effects of BTS treatment on structural alterations in *Sapje* (*Sap*) and *Candyfloss* (*Caf*) larval muscle

**Fig 1:**
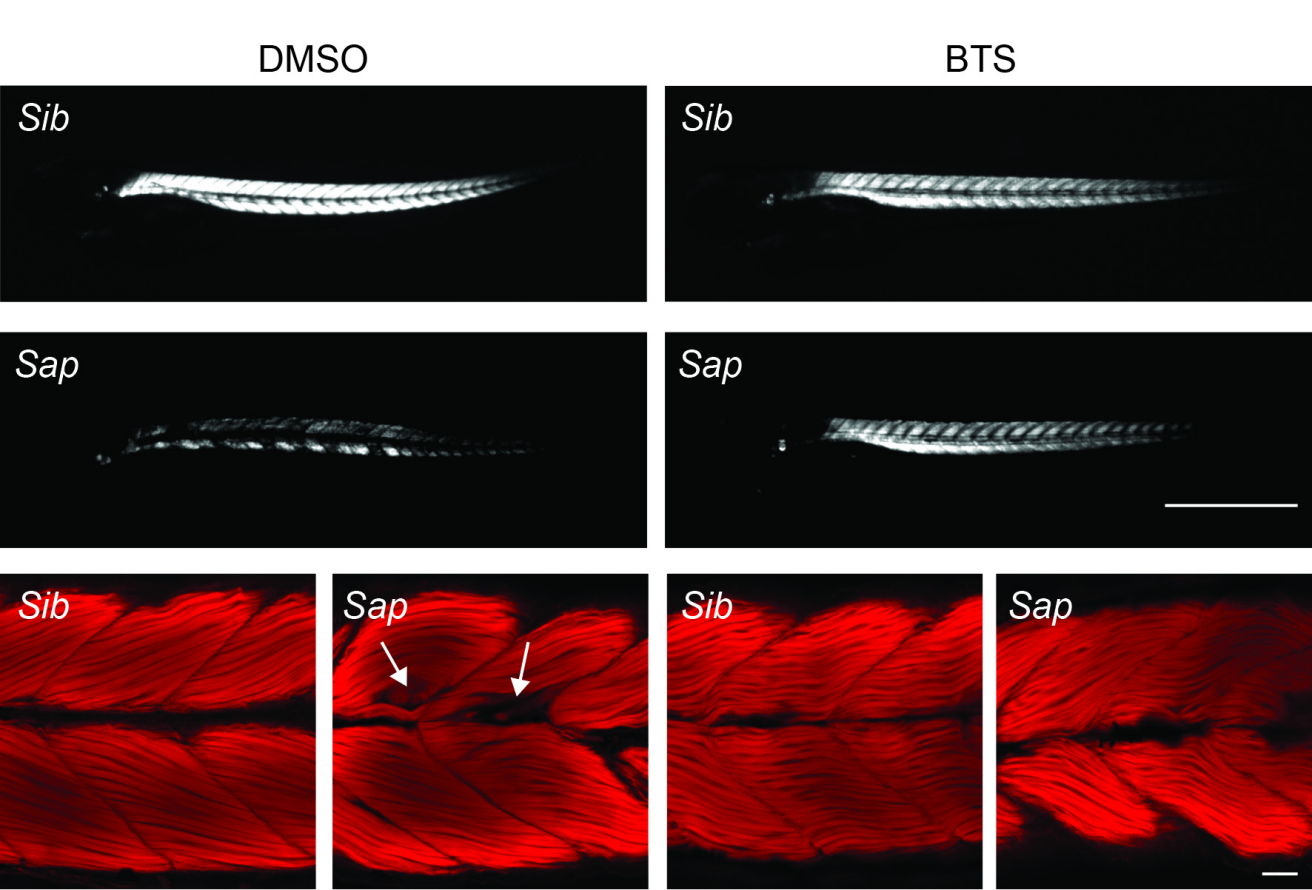
Early immobilization by BTS prevents muscle structural changes in *Sapje* (*Sap*) mutant larvae and their normal siblings (*Sib*) at 4 dpf. Upper panels: birefringence microscopy of *Sap* and siblings from 0.1% DMSO and 50 μM BTS treatment respectively. Lower panels: whole mount preparations with Rhodamine phalloidin staining. Arrows indicate altered areas. These illustrations were typical of n = 6 Rhodamine phalloidin stained samples per each group, in 3 treatment sets. Scale bars: 1 mm (upper); 50 μm (lower).

**Fig 2:**
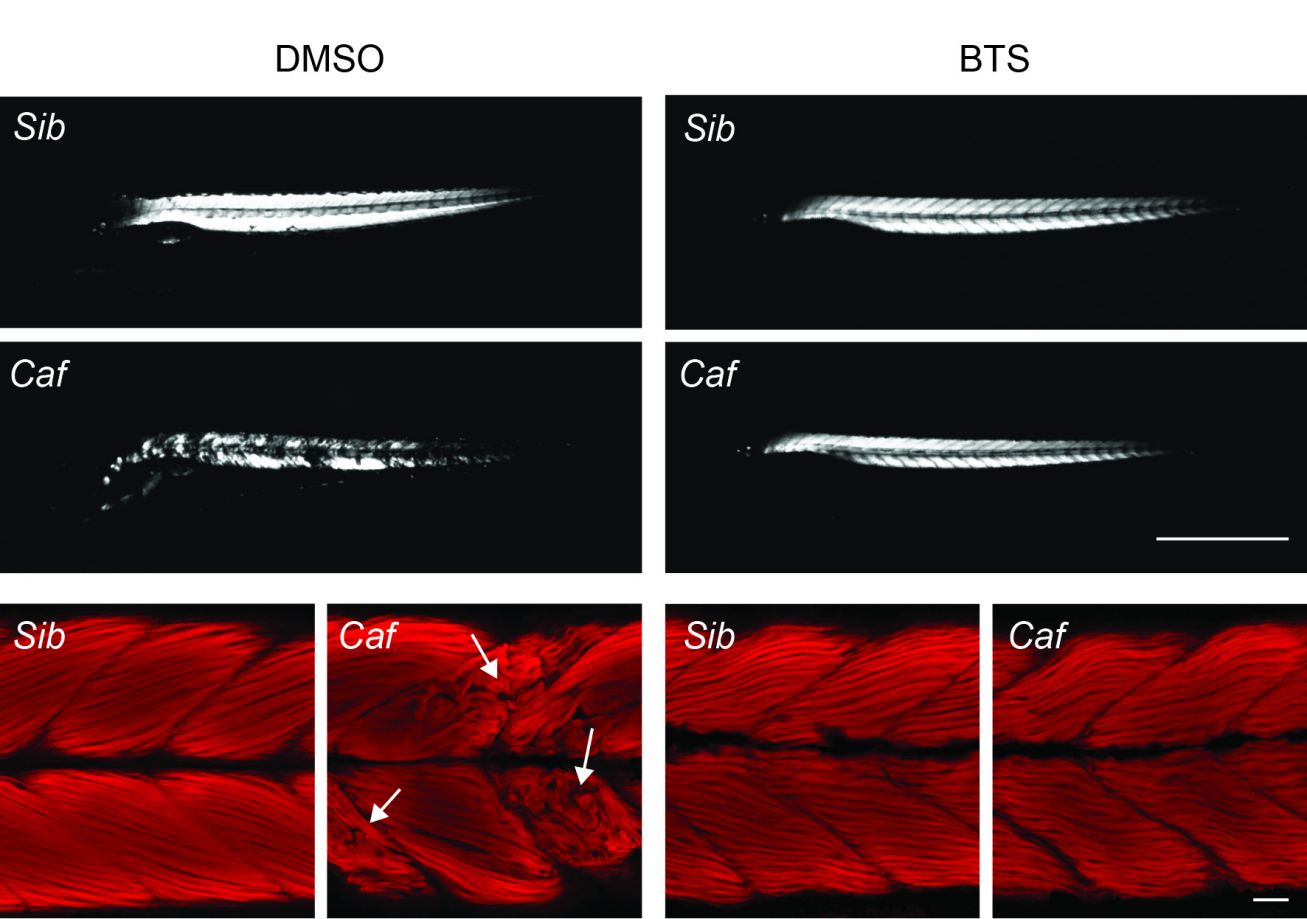
Early immobilization by BTS prevents muscle structural changes in *Candyfloss* (*Caf*) mutant larvae and their normal siblings (*Sib*) at 4 dpf. Upper panels: birefringence microscopy of *Caf* and siblings from 0.1% DMSO and 50 μM BTS treatment respectively. Lower panels: whole mount preparations with Rhodamine phalloidin staining. Arrows indicate altered areas. These illustrations were typical of n = 6 Rhodamine phalloidin stained samples per each group, in 3 treatment sets. Scale bars: 1 mm (upper); 50 μm (lower).

**Fig 3:**
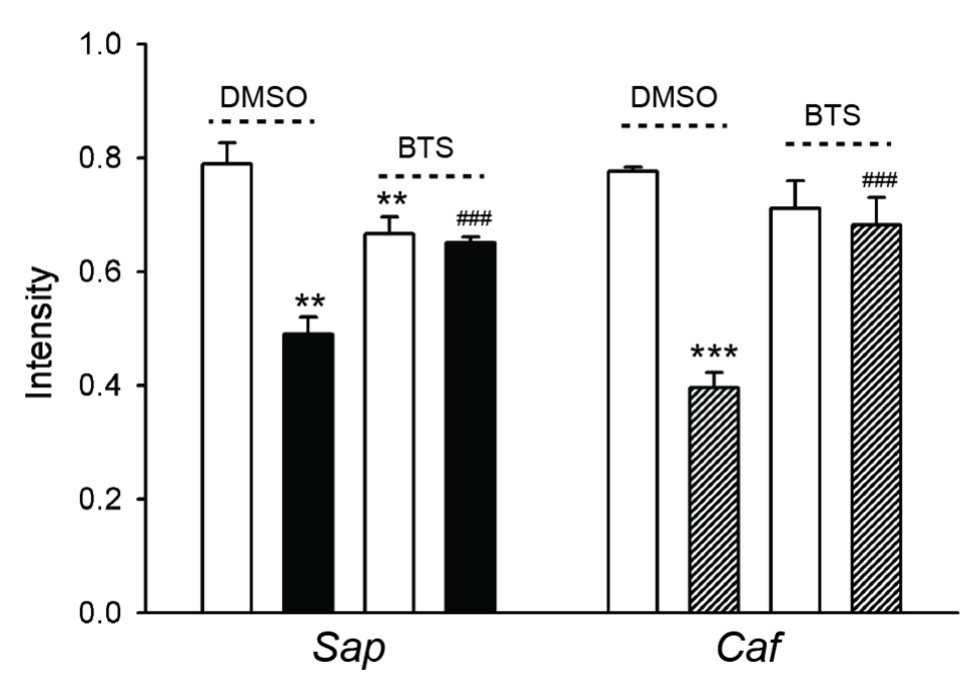
Quantification of birefringence intensity in normal siblings (*Sib*, open bars) and mutated larvae of *Sapje* (*Sap*, filled bars) and *Candyfloss* (*Caf*, hatched bars), at 4 dpf. Larvae were treated with 0.1% DMSO or 50 μM BTS. n = 3–4 in each group. **, p< 0.01 *** p< 0.001 compared to the *Sib* in DMSO, ###, p<0.001 compared to the mutants (*Caf* or *Sap*) in DMSO (ANOVA, Holm-Sidak method). No significant difference was detected between the BTS treated groups.

Figs 1 and 2 show birefringence microscopy of 4 dpf *Sap* and *Caf* larvae treated with DMSO or BTS. As seen in the photographs, normal siblings from both strains exhibited uniform light pattern under polarized light, while the DMSO control *Sap* and *Caf* mutant larvae displayed significantly reduced and irregular birefringence with patchy areas in the musculature. Following BTS treatment, the birefringence pattern was still strong in the normal *Sap* and *Caf* siblings, but appeared somewhat weakened (Figs 1 and 2, top panels). In contrast, the birefringence signal of *Sap* and *Caf* mutant larvae was markedly improved (Figs 1 and 2, middle panels). To further investigate the structural alterations, the larval preparations from these experimental groups, where birefringence patterns were recorded, were fixed and stained with Rhodamine phalloidin. As seen in the lower panels of Figs 1 and 2, both DMSO control *Sap* and *Caf* larvae exhibited generally disorganized muscle structure (arrows) compared to their siblings. We observed areas with gaps in the musculature and wavy distorted muscle fibers. After BTS immobilization, the structure of both *Sap* and *Caf* larval muscles shows more regular fiber arrangements and sarcomere patterns, reflecting an improved muscle organization. However, all BTS treated groups, the *Sap*, *Caf* mutated larvae and their siblings, had developed muscles with somewhat wavy arrangement of the muscle compared to the DMSO groups. The results from these experiments thus show that the beneficial effects of BTS treatment in *Sap* and *Caf* larvae are seen in both Rhodamine phalloidin stained samples and birefringence examinations. In addition, both birefringence and Rhodamine phalloidin staining of siblings suggest that BTS treatment/immobilization introduces structural alterations in the musculature. As shown in Table 1, the whole body length and body width (around somite number 7) were similar in the DMSO control *Sap* and *Caf* groups compared to their siblings, and remained unchanged in all groups after BTS treatment. To quantify the extent of muscle structural alteration and the improvement after BTS treatment, we evaluated the birefringence by an optical method where the relative amount of light areas in the muscle (i.e. the areas with normal birefringence) was quantified. The summarized data in Fig 3 shows a significantly lower birefringence signal in the *Sap* and *Caf* mutant groups and an improvement after BTS treatment. The data thus demonstrate significant structural abnormalities in the *Sap* and *Caf* mutated groups compared to their siblings, and that these changes were significantly reduced by BTS immobilization. All sibling groups were, however, also influenced by the BTS treatment giving a partly reduced birefringence signal (open bars in Fig 3) and the appearance of a wavy arrangement of the muscle fibers (Figs 1 and 2). The extent of the “waviness” in the Rhodamine phalloidin stained samples was evaluated using line tracking. The ratio of cell length to myoseptal length was 1.04±0.01 in normal siblings (3 *Caf* and 3 *Sap* siblings) suggesting an almost straight arrangement. This value increased (p<0.001) to 1.13±0.01 after BTS treatment, reflecting a more wavy organization. Similar results were found in the BTS treated mutant larvae (*Sap*: 1.05±0.01, + BTS: 1.13±0.01, p<0.001; *Caf*: 1.03±0.01, + BTS: 1.16±0.01, p<0.001). These results thus show that BTS immobilization results in a loss of the straight alignment of myofibers in all groups.

**Table 1:** General development of zebrafish 4 dpf larvae from *Sapje* and *Candyfloss* mutants (*Sap*, *Caf*) and from their normal siblings (*Sib*), after incubation with 0.1% DSMO or 50 μM BTS; n = 3–5 in each group.

0.1% DMSO					50 μM BTS			
	*Sap*	*Sap Sib*	*Caf*	*Caf Sib*	*Sap*	*Sap Sib*	*Caf*	*Caf Sib*
Body Length (mm)	3.51±0.03	3.40±0.02	3.42±0.03	3.45±0.02	3.45±0.03	3.48±0.03	\3.45±0.01	3.44±0.03
Body Width (mm)	0.221±0.005	0.221±0.002	0.226±0.006	0.225±0.005	0.219±0.004	0.218±0.002	0.220±0.003	0.223±0.003

### Effects of BTS treatment on muscle function in *Sap* and *Caf* larval muscle

**Fig 4:**
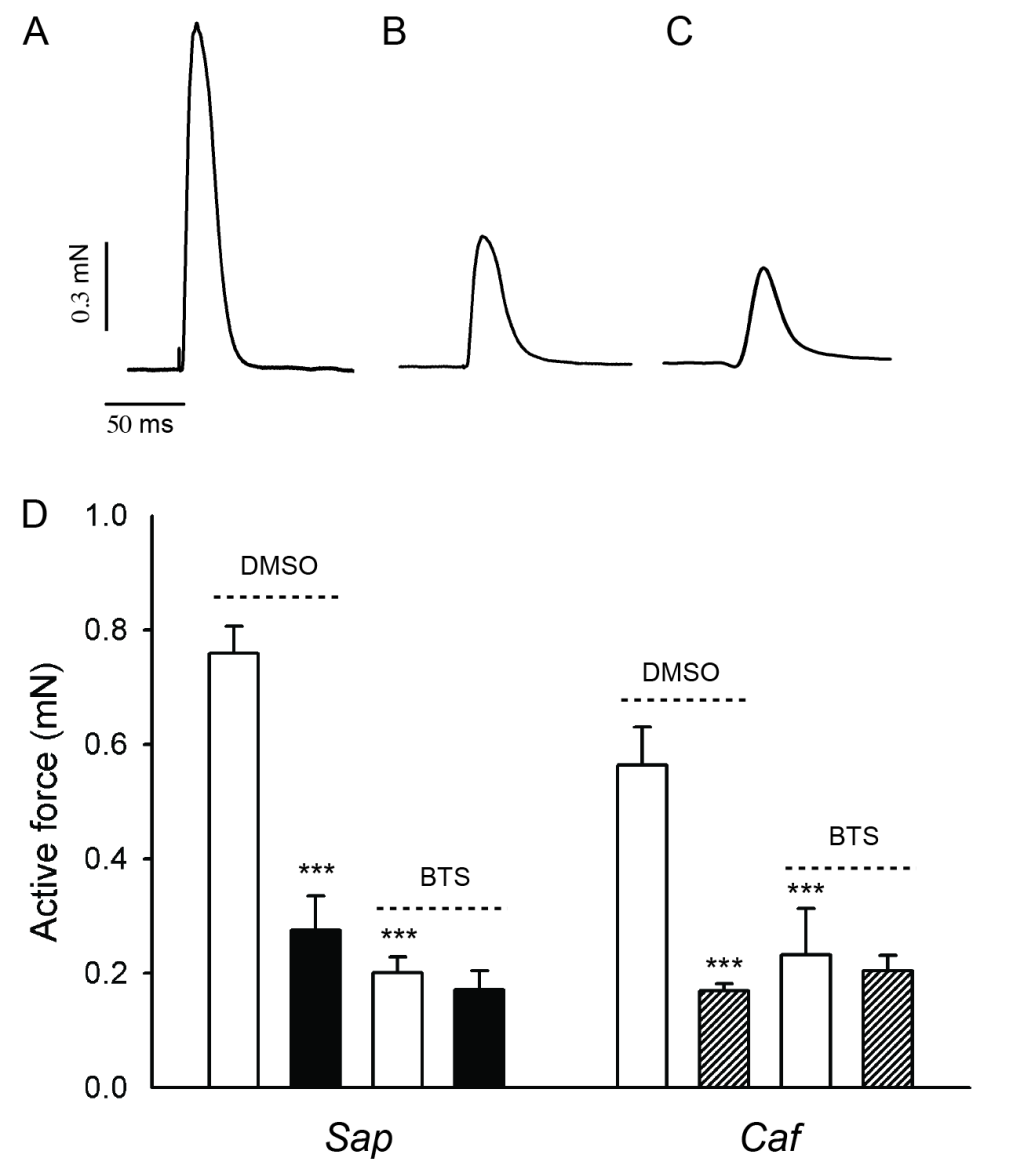
Active force measurements from *Sapje (Sap)*, *Candyfloss (Caf)* mutant larvae and their siblings (*Sib*) at 4 dpf. Panel A -C show representative force traces of single twitch contraction from a sibling, *Sap* and *Caf* mutant larva respectively. Panel D shows the summarised data from 4 dpf *Sap* (filled bars), *Caf* mutant (hatched bars) and *Sib* larvae (open bars) with, or without BTS immobilization. ***, p<0.001 compared to the *Sib* in DMSO (ANOVA, Holm-Sidak method). No significant difference was detected between the BTS treated groups. n = 4–10 in *Sib* groups; n = 3–5 in *Sap* and *Caf* groups.

We characterised the function of the *Sap* and *Caf* muscles after DMSO and BTS treatment. The larvae were held and immobilized in 50 μM BTS from 18 hpf to 4 dpf, or freely moving in the 0.1% DMSO control medium. After washout of BTS in normal E3 medium for 1 hour, the larvae started to move, showing reversibility of the immobilization. The larvae were then euthanized and mounted for mechanical measurements at 4 dpf. Single twitch stimulation was applied and the muscles were stretched to a length giving maximal active force. The upper panels (A-C) of Fig 4 show original force traces from 4 dpf muscle preparations of a *Caf* sibling, a *Sap* and *Caf* mutant larvae from the DMSO control group. The two latter muscle preparations were markedly weaker. Panel D of Fig 4 summarizes the active force data showing a significantly weaker active force in *Sap* and *Caf* larvae compared to their siblings. After BTS immobilization, the siblings, *Sap* and *Caf* muscles were all weaker compared to the corresponding DMSO treated groups. However, the difference between the mutated strains and their siblings was absent. These findings show that both *Sap* and *Caf* mutations result in a significant contractile impairment, and that the immobilization by BTS generally weakens the skeletal muscles at 4 dpf. However, the immobilization also removes the difference in active force between the mutated strains and their siblings.

**Fig 5:**
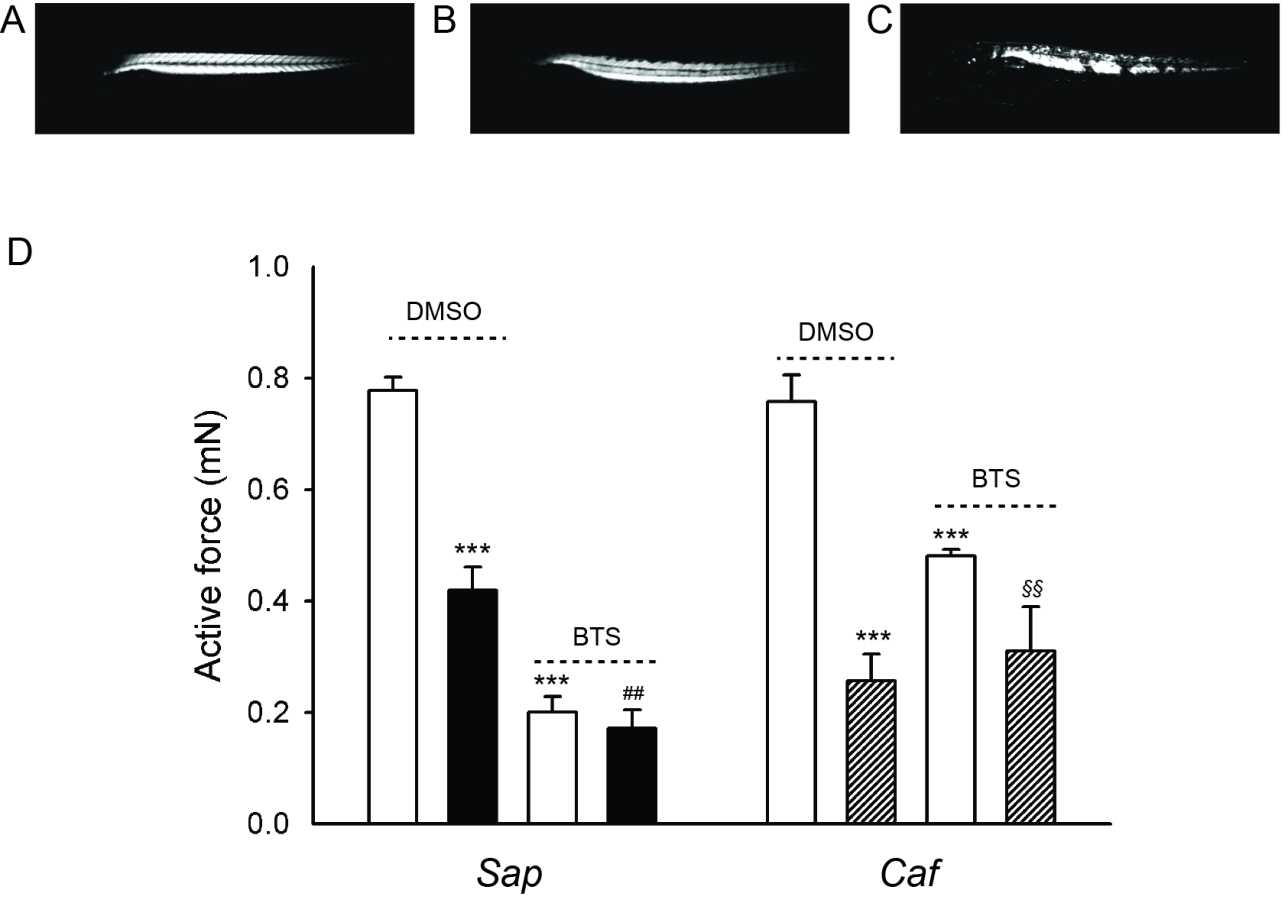
Recovery in E3 medium after BTS immobilization induces structural changes in *Candyfloss* (*Caf*) larvae at 5 dpf. Panel A-C shows birefringence microscopy of a Sibling (*Sib*), *Sapje* (*Sap*) and *Caf* larva from one-day E3 medium recovery. Panel D summarizes the corresponding force measurements of two mutant strains at 5dpf. *Sap*: filled bars; *Caf*: hatched bars; *Sib*: open bars. ***, p<0.001 compared to the *Sib* in DMSO, ##, p<0.01 compared to the *Sap* in DMSO, §§, p<0.01 compared to the *Sib* in BTS (ANOVA, Holm-Sidak method). No significant difference was detected between the BTS treated *Sap* groups. n = 3–10 in *Sib* groups; n = 3 in *Sap* and *Caf* groups.

Since treatment with BTS reduced the structural changes as measured with birefringence in the mutated strains, but also impaired contractile function in general at 4 dpf after one-hour washout, we explored if a longer period of active movement after BTS treatment would improve contractility of the sibling muscles, and cause structural changes in the mutants. We thus held larvae following BTS treatment (as used in the results described above) in E3 medium for one day until 5 dpf and analyzed mechanical function as shown in Fig 5. We observed that the *Caf* normal siblings appeared to be more active, with longer burst of swimming compared to the *Sap* normal siblings. Interestingly, the active force of this group also recovered better compared to the *Sap* siblings after the immobilization period. The BTS treated *Caf* siblings gave about 40% of the DMSO group at 4 dpf (Fig 4) and recovered to about 64% at 5 dpf (Fig 5). The corresponding values for the *Sap* siblings were 27% and 26% (Figs 4 and 5). At 5dpf, the *Sap* larvae did not reveal any birefringence alterations (Panel B, Fig 5) and gave similar active force as their siblings (left diagram, Panel D). In contrast, the *Caf* larvae at 5dpf were weaker than their siblings (right diagram, Panel D, Fig 5) and developed structural changes with patchy alterations in birefringence (Panel C, Fig 5). These data suggest that active movement improves the mechanical function after BTS immobilization in the *Caf* sibling group, but also introduces structural changes in the *Caf* mutants.

### Inter-strain difference between *Sap* and *Caf* mutants

The structural/functional alterations in *Sap* and *Caf* dystrophic mutants, and the protective effects of immobilization at 4 dpf were all similar between the two strains. However, the observation at 5 dpf, after one-day washout of BTS, revealed a bigger vulnerability in the *Caf* mutants compared to the *Sap* group. To investigate if any general difference present between the two models that could explain the divergent at 5 dpf, behavioural analysis was performed on the two mutant strains. As summarised in Table 2, the overall active swimming time tended to be more in the *Caf* strain, possibly suggesting a more active behaviour of the *Caf*. Interestingly, the moving speed was similar in all groups.

**Table 2:** Swimming activity of zebrafish 5 dpf larvae from *Sapje* and *Candyfloss* mutants (*Sap*, *Caf*, *n = 4–5*) and from their normal siblings (*Sib*, *n = 20–21*). All larvae were monitored for 2 minutes at room temperature.

	*Sap Sib*	*Sap*	*Caf Sib*	*Caf*
Active time %	55±4	55±9	62±2	57±8
Speed (cm/s)	2.1±0.2	1.7±0.3	2.0±0.1	1.2±0.2

## Discussion

We show that normal muscular activity *in vivo* is a main factor for initiating structural changes in both dystrophin and laminin α2-chain associated muscular dystrophy in the zebrafish larvae. We also demonstrate that laminin α2-chain deficiency in the zebrafish leads to a significant muscle weakness, associated with structural alterations, similar to those previously described in the dystrophin-null zebrafish larvae [21].

The dystrophy models examined in the study reflect alterations in the intracellular link of the DGC complex to the cytoskeleton and in its extracellular coupling of the matrix. Both of these alterations give rise to severe muscular dystrophy in humans, the Duchenne (DMD) and the laminin α2-chain/merosin (MDC1A) dystrophy. We found that the *Caf* mutant larvae, in a similar manner as the *Sap* mutants, have a significant contractile dysfunction associated with the structural damage. These two zebrafish models thus reproduce several of the structural and functional characteristics of human muscular dystrophy. Furthermore, the zebrafish enables a unique set of experiments compared to the young adult and adult mouse models, since *in vivo* challenges of skeletal muscle in the zebrafish models can be introduced without major global secondary effects on the animal physiology, as they are not dependent on food intake, respiratory function or heart beating at the larval stage.

We applied a pharmacological approach to inhibit cross-bridge cycling and muscle contraction. BTS has been identified as a specific blocker of actin-activated ATPase of fast myosin [26], and we have previously shown that BTS inhibits zebrafish larval muscle *in vitro* [25]. We thus consider BTS to abolish contraction in the larvae via direct effects on the contractile system, without interfering with motor nerves, muscle activation or imposing external constraints. This would inhibit effects of active contraction on the membrane strain in the muscle, and possibly also the disease progression caused by contraction-induced membrane strain in the two dystrophic models. Since the defect in both the *Sap* and *Caf* models originates in mutations at the genomic level, it is very unlikely that BTS treatment or immobilization could cause any recovery of dystrophin or laminin α-2 expression. We applied a long-term treatment with 50 μM BTS, a concentration used in previous studies in myotubes [29]. The BTS treatment resulted in a significant inhibition of active force that persisted for at least one day after washout in E3 medium. Since the inhibitory effect of the compound is rapidly reversible (present study, Dou *et al*. 2008 [25]), we expect that the lower active tension after BTS treatment is the result of an impaired establishment of functional sarcomeres. This is consistent with results from BTS treated *Xenopus* cultured myotubes [29] and zebrafish immobilized with Tricaine [30], showing that active cross-bridge cycling is an important component in myofibrillogenesis. Active force was about 27–40% of control following BTS treatment in normal larvae siblings. Since the birefringence intensity and muscle size were not markedly reduced after BTS treatment, the lower force cannot be explained by loss of muscle mass or sarcomere assembly, but rather by an internal change in the myofibrillar bundling as seen after short-term Tricaine immobilization [30]. It is important to note that, since active cross-bridge cycling is an important component of muscle development, all factors affecting active force production will result in additional effects on the structure of the contractile machinery. The mechanical sensor and cell signalling involved remain to be elucidated.

A key finding of this study is that the BTS immobilization reduced the muscle damage occurring in freely moving *Sap* and *Caf* larvae. Although BTS treatment generally weakened muscle function, as discussed above, it also abolished the difference in force between the mutated larvae and their normal siblings. These results show that active contraction is an important initiating mechanism in the pathogenesis of the dystrophy in these zebrafish models.

We find that BTS immobilization affected the structural damage of both the *Sap* and *Caf* mutant muscles. This suggests that both models, associated with alterations in components of the sarcolemmal attachments, have similar sensitivity to mechanical stress. This finding, from an *in vivo* immobilization, thus shows that the normal activity of the living animals including swimming is sufficient to induce the structural lesions. In a previous study on mice, it was found that laminin α2-chain deficiency did not affect the acute response to stretch [14], which might suggest that a longer period of activity is required for the structural damage. Interestingly, we find that the *Caf* sibling larvae had generally higher swimming activity than the *Sap* siblings. This most likely reflects a subtle difference in animal behaviour between the two strains. The *Caf* mechanically recovered better than the *Sap* larvae under the same condition suggesting that activity is an important component in the muscle recovery after immobilization. Structural damage was observed in the *Caf* mutants after BTS washout showing that one day of physical movement is sufficient to induce a structural damage in this model. The less active *Sap* siblings did not recover muscle force within one day after BTS washout, and did not show structural damage. It is thus possible that differences in muscle damage development after BTS removal between *Sap* and *Caf* reflects changes in the motor behaviour. However, other strain dependent factors, and most likely the impact of the respective mutations on the injury susceptibility of muscle, also contribute to the difference between *Caf* and *Sap*.

In conclusion, we show that the *Caf* zebrafish mutant larvae have a significant mechanical dysfunction associated with the structural defects. Inhibition with BTS results in a weaker contractile performance, most likely due to delayed/altered development of the contractile system. However, in both types of muscular dystrophy, dystrophin or laminin α2-chain deficiency, early mechanical silencing *in vivo* prevents the onset of the structural alterations, consistent with a membrane challenge due to normal physical activity being a major contributing factor in the development of muscular dystrophy.

## Supporting Information

S1 Data Data for Figures and Tables. (XLSX)
